# Microbial lipid production by oleaginous yeasts grown on *Scenedesmus obtusiusculus* microalgae biomass hydrolysate

**DOI:** 10.1007/s00449-020-02354-0

**Published:** 2020-04-28

**Authors:** Samer Younes, Felix Bracharz, Dania Awad, Farah Qoura, Norbert Mehlmer, Thomas Brueck

**Affiliations:** Werner Siemens-Lehrstuhl für Synthetische Biotechnologie, Technische Universität München, Garching, Germany

**Keywords:** *Scenedesmus obtusiusculu*, *Cutaneotrichosporon oleaginosus*, Enzymatic hydrolysis, Microalgae biomass, Lipid production

## Abstract

**Abstract:**

Due to increasing oil prices and climate change concerns, biofuels have become increasingly important as potential alternative energy sources. However, the use of arable lands and valuable resources for the production of biofuel feedstock compromises food security and negatively affect the environment. Single cell oils (SCOs), accumulated by oleaginous yeasts, show great promise for efficient production of biofuels. However, the high production costs attributed to feedstocks or raw materials present a major limiting factor. The fermentative conversion of abundant, low-value biomass into microbial oil would alleviate this limitation. Here, we explore the feasibility of utilizing microalgae-based cell residues as feedstock for yeast oil production. We developed an efficient, single‐step enzymatic hydrolysis to generate *Scenedesmus obtusiusculus* hydrolysate (SH) without thermo-chemical pretreatment. With this eco-friendly process, glucose conversion efficiencies reached 90–100%. *Cutaneotrichosporon oleaginosus*, *Cryptococcus curvatus* and *Rhodosporidium toruloides* were cultivated on SH as sole nutrients source. Only *C. oleaginosus* was able to accumulate intracellular lipids, with a 35% (g lipid/g DCW) content and a yield of 3.6 g/L. Our results demonstrate the potential valorization of algal biomass into desired end-products such as biofuels.

**Graphic Abstract:**

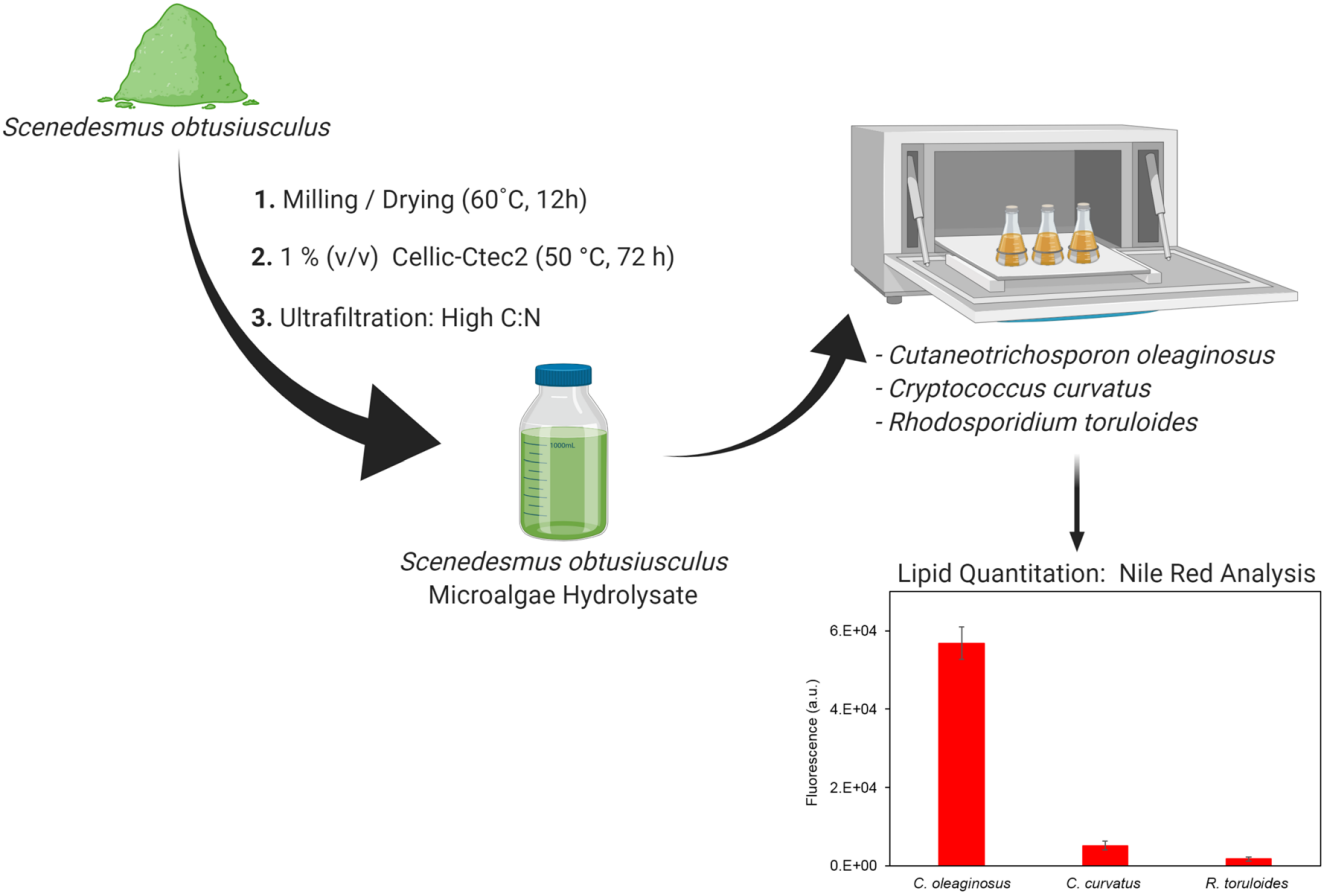

## Introduction

The ever-increasing energy demand in today’s industrial world led to the widespread use of non-renewable fossil fuels such as petroleum. The transition from a society with waste generating, linear production routes to one cyclic valorization path in conjunction with renewable resource management is one of the most demanding technological goals for establishing sustainable bioeconomy [1, 2]. This scenario particularly applied renewable energy supply routes that demand a switch from finite fossilto sustainable platform solutions. Moreover, dwindling of fossil resources, escalating environmental pollution, surging CO_2_ and greenhouse gas emissions, in addition to climate change have collectively driven the search for alternative energy sources [3]. Accordingly, technological innovations that enable a more sustainable lifestyle are coveted [4].

Biofuels have garnered great interest in recent years as alternatives for fossil fuel. In fact, plant-derived biofuel offers a partial solution to the ever-increasing energy demand, due to their renewability. However, this first-generation of biofuels, generated from edible crops, impacts agricultural activity and jeopardizes food security [1] To meet the current annual global-demand of biodiesel, more than double of the currently arable land would be required to grow crops that are explicitly grown for fuel production [5]. Consequently, alternative sources for biofuel production that do not affect food security are in high demand [1]. One of those alternatives is the use of oleaginous microorganisms such as algae and yeast [6].

Oleaginous microorganisms accumulate lipid at a minimum of 20% (g lipids/g dry cell weight (DCW)) [7]. However, lipid accumulation in oleaginous microorganisms, such as yeast, fungi and microalgae, is not a constitutive feature, but rather an adaptive response to particular environmental factors [8]. In environmental conditions abundant in carbon source and deficient in specific nutrients such as nitrogen, phosphorus or sulfur, oleaginous microorganisms convert excess carbon into fatty acids and incorporate them into triglycerides (TAGs) as a form of energy storage [9]. TAGs are stored in specialized organelles called lipid bodies (also known as lipid droplets) [10]. Single Cell Oils (SCOs) can be efficiently converted into biodiesel and biofuel [11, 12]. Various oleaginous yeasts have been subject to extensive investigations such as *Yarrowia lipolytica*, *Rhodosporidium toruloids* and *Lipomyces starkeyi*, with reports of lipid accumulation in excess of 70% (g lipid/g DCW) [13, 14]. The potential biotechnological applications of these oleaginous yeasts, utilizing various carbon sources have been previously reported [9, 15, 16].

Moon et al. first isolated *Cutaneotrichosporon oleaginosus* (ATCC 20509) in 1978 from factory drain samples of the Iowa State University Dairy Farm. *C. oleaginosus* readily utilizes glucose, galactose, cellobiose, xylose, sucrose, and lactose as carbon source [1, 17–19]. Furthermore, this yeast is able to metabolize glycerol, *N*-acetylglucosamine, volatile fatty acids and ethanol and 4-hydroxymethylfurfural [20–22]. To improve the sustainability of SCOs from socio-economic aspects, *Y. lipolytica* has undergone extensive genetic engineering aimed at simultaneous sugar uptake (hexoses and pentoses) from complex and wastewater hydrolysates, which is an inherent ability in *C. oleaginosus* [8]. Depending on carbon, nitrogen sources and stress conditions (nitrogen, phosphate or sulfate limitation), cellular lipid accumulation in *C. oleaginosus* can reach up to 85% (g lipid/g DCW) [23, 24]. In addition to fast growth rate, this oleaginous yeast exhibits a fatty acid profile that mimics that of vegetable oils, specifically palm oil, with palmitic, stearic and oleic acid as dominant fatty acids [25]. McCurdy et al. reported that biodiesel B20 derived from *C. oleaginosus* TAGs meet the ASTM (D6751) certification [1, 9]. Through recycling and finding appropriate industrial sink for bio-compounds, a recent study touching on the socio-economic sustainability of *C. oleaginosus* SCOs has been recently prepared in our group [4].

Other oleaginous species that can be exploited for the biofuel sector include microalgae. In contrast to industrial crops, such as palm or canola plants, biomass generation from microalgae has high space and time yields. Globally, around 280 tons/ha of algae dry biomass and 3.9 tons/ha of forest biomass are produced every year [26]. Additionally, microalgae display high CO_2_ fixation ability (513 tons of sequestered CO_2_ per hectare per year). Specifically, 1.6-2 grams of CO_2_ is captured for every gram of algal biomass produced, at an efficiency of 80-99% [27, 28]. While microalgae can provide renewable oils by photosynthetically converting atmospheric CO_2_ to lipids, yields are conventionally lower compared to oleaginous yeast species [29–31]. In most algae oil production processes, the extracted cell residue is not contributing to the overall process economy [29, 31].

Yet recently, several value-adding outlets for this residue have been achieved, either by feeding it back into renewable production (oil, food, feed, etc.) or by recycling of resources [4, 32]. Similar waste-free biorefinery approaches have been considered in the design and optimization of biogas production processes [33]. In that respect, the residual biomass, which is rich in fermentable sugars, can be used as feedstock for oleaginous yeasts cultivation [34]. Specifically, *Scenedesmus* spp. belong to the most common freshwater green algae. *Scenedesmus obtusiusculus* A189, a newly isolated member of the *Chlorophyta* genus, is characterized by high growth rates in fresh and saline media, in addition to high inherent carbohydrate content [35]. Fermentative conversion of various biomass sources into SCOs and subsequent biofuel via oleaginous yeasts has recently received an increasing interest in the scientific and industrial community [2, 4]. Specifically, microalgae biomass does not contain recalcitrant lignin, making it suitable for eco-friendly and cost-effective hydrolysis methods [35]. To that end, efficient hydrolysis of algae biomass could provide a sustainable stream of monomeric hexose and pentose sugars for fermentative growth. However, chemical biomass hydrolysis, which is most commonly applied in industry, tarnishes the eco-friendly aspect of biofuel production [2]. Alternatively, enzymatic hydrolysis efficiently generates sugar-rich fermentation media, yet necessitates thermo-chemical pretreatment steps to break down the lignocellulosic biomass components into monomeric hexose and pentose sugars [36]. These pretreatment steps release inhibitory compounds that hinder growth of subsequent oleaginous yeast inoculum in the prospective hydrolysate. Removal of these inhibitory compounds imposes additional costs, time and effort for the production process [37].

In this study, we examine the enzymatic hydrolysis of microalgae-based cell residues and the subsequent use of its hydrolysate (SH) for the cultivation of oleaginous yeasts (*C. oleaginosus*). A single-step enzymatic hydrolysis process was devised and optimized, allowing efficient hydrolysis and saccharification of microalgae biomass, without thermo-chemical pretreatment. The resulting *S. obtusiusculus* hydrolysate (SH), barring expensive additives, was utilized as the sole fermentative media for *C. oleaginosus*, *Cryptococcus curvatus* and *Rhodosporidium toruloides*. The accumulated lipids, deposited as intracellular lipid bodies (LBs), were relatively quantified by Nile red analysis. Moreover, *C. oleaginosus* growth, dry biomass and lipid weight were evaluated.

## Materials and methods

### Algae strains and biomass determination

*Scenedesmus obtusiusculus* (A189) residues were obtained from Pharmaceutical Biology Group, Ernst Moritz Arndt University (EMAU), Griefswald, Germany. Water content was determined following the milling and drying of the algal samples at 60 °C overnight. Total carbohydrate concentration was determined by the thymol-sulfuric acid method [38]. The standard Kjeldahl procedure was utilized to determine the amount of protein in the algae biomass [39]. Total lipids were extracted according to Folch et al., and determined gravimetrically after solvent evaporation [40]. Biomass ash content was determined by following the AOAC procedure [41]. Biological replicates ensured reproducible measurements.

### Enzymatic hydrolysis and preparation of SH

*Scenedesmus obtusiusculus* Hydrolysate (SH) was prepared by hydrolyzing autoclaved algae biomass. Briefly, dry biomass samples weighing 50 g were transferred to 2 L glass bottles containing 1 L of 50.0 mM sodium acetate buffer, pH 5.0. Different hydrolytic enzyme mixtures were examined, including Cellic-Ctec2 (Novozymes, Denmark), Celli-Htec2 (Novozymes, Denmark), Pectinex (Novozymes, Denmark) and Fungamyl (Novozymes, Denmark). Hydrolytic Reactions were initiated by adding the enzyme solution and incubating the mixture at 50 °C for 72 h. Buffer and enzymes were sterile-filtered prior to hydrolysis. Samples were then spun down for 30 min. Cross-filtration using a 10 kDa membrane made from regenerated cellulose was completed under the following parameters: Inlet-Pressure (P1) of 2 bar, Repentant-Pressure (P2) of 0.3–0.5 bar and permeate was open to atmospheric pressure. Flow-Rates of retentate and permeate were adjusted to 2 L/min and 0.1 L/min respectively. A 0.2 µm filter capsules were installed at the outlet to sterilize the resulted hydrolysate. Biological triplicates of the SH were prepared.

### Sugar analysis

Sugar composition of the hydrolysate was determined by an Agilent 1100 series HPLC with a Refractive Index (RI) detector (Shodex, RI101) and Ultraviolet Index (Sedere-France, Sedex75). Following cross-filtration, 5 µL sample was injected on an Aminex HPX-87P column (8% cross-linked resin, lead ionic, Bio-Rad) and separated at 70 °C with double-distilled water as mobile phase. Run parameters were set to a duration of 30 min, a flow rate of 0.4 mL/min and detection at 50 °C. Samples’ RI signal was aligned with that of internal standard curves.

### Yeast strains and culture conditions

Yeast strains *Cutaneotrichosporon oleaginosus* (ATCC 20509), *Cryptococcus curvatus* (CBS 5324) and *Rhodosporidium toruloides* (NP11) were maintained on yeast peptone dextrose (YPD) agar (20 g/L peptone, 10 g/L yeast extract, 20 g/L glucose, 20 g/L agar) at 4 °C for short-term storage. Minimal nitrogen media (MNM) (30 g/L glucose, 1.5 g/L yeast extract, 0.5 g/L NH_4_Cl, 7.0 g/L KH_2_PO_4_, 5.0 g/L Na_2_HPO_4_·12H_2_O, 1.5 g/L MgSO_4_·7H_2_O, 0.08 g/L FeCl_3_·6H_2_O, 0.01 g/L ZnSO_4_·7H_2_O, 0.1 g/L CaCl_2_·2H_2_O, 0.1 mg/L MnSO_4_·5H_2_O, 0.1 mg/L CuSO_4_·5H_2_O, 0.1 mg/L Co(NO_3_)_2_·6H_2_O; pH 5.5) was adopted from Suutari et al. for induction of lipogenesis [42].

Following pre-culturing in YPD broth for 24 h, yeast cells were centrifuged, washed with PBS buffer (8 g/L NaCl, 0.2 g/L KCl, 1.44 g/L Na_2_HPO_4_, 0.24 g/L KH_2_PO_4_; pH 7.4) and inoculated in 250 mL baffled flasks, containing 50 mL of MNM, at an initial seeding OD_600_ of 0.5. Incubation lasted for 4 days in a rotary shaker at 120 rpm and 28 °C. To evaluate the capability of utilizing hydrolysates for yeast growth and lipid accumulation, the selected oleaginous strains were cultured solely on SH lacking any additives or carbon supplementation. Biological cultures of the oleaginous yeasts were carried out in triplicates.

### Nile red staining

Samples were analyzed in technical triplicates using a modified protocol from Sitepu et al. [43]. Briefly, 225 µL of each yeast culture was transferred to a 96-well black microtiter plate. Serial dilutions were performed in triplicates to ensure an optical density < 1 before 50 µl DMSO was added to each well. Initial absorbance readings were taken at 600 nm and for growth monitoring and correction of fluorescence readings for growth variation. A volume of 25 µL Nile red was then added to each well (final concentration of 50 µg/mL). Fluorescence measurements (recorded before and after Nile red addition) at excitation at 530/25 nm; emission at 590/35 nm; and kinetic reading for 5 min with 30 s interval were taken. Maximal emission values were determined and fluorescence measurements were corrected for variation in cell density by dividing the fluorescence unit by background optical density OD_600_ values.

### Gravimetric analysis

Technical triplicates of the total lipid content of the oleaginous yeasts were determined by a modified method by Folch et al. [40]. Briefly, 15 mL of yeast cultures were washed and homogenized using an Avestin Emulsiflex at a sample port pressure of 1200 bar and a chamber pressure of 8 bar. Lipids from the homogenate were extracted with 6 mL of Folch solution (2:1 chloroform: methanol). Lipid extraction continued overnight at room temperature and shaking speed of 120 rpm. Subsequently, 1.2 mL of 0.9% NaCl were added to aid phase separation. The lower phase was aspirated using a syringe and added to pre-weighed glass vessels. The chloroform was fully evaporated under a nitrogen stream and glass vials were weighed again. The extracted lipid samples were used to calculate lipid content as total lipid weight and as percent of dry yeast weight.

### Dry biomass determination

A volume of 2 mL of each yeast culture was transferred in triplicates to pre-weighed 2 mL Eppendorf tubes. The tubes were weighed again the following centrifugation at 14,000 g for 5 min, washing and drying at 60 °C overnight. Measurements were recorded in triplicates by subtracting the weight of the sample tubes from their respective pre-weights.

## Results

### Algae biomass analysis

In this study, the biomass composition of *S. obtusiusculus* was determined (Table 1). This green algae displays high amounts of carbohydrates and crude proteins comprising 34% and 49% (g/g DCW) respectively. Other components encompassed water, lipids and ash measured at 3.7, 8.3 and 1.9% (g/g DCW), respectively. The acquired biomass data suggest that *S. obtusiusculus* can be quantitatively hydrolyzed by chemical and enzymatic systems to release monomeric pentose and hexose sugars, which could serve as a carbon source for microbial cultivation.

**Table 1:** Biochemical composition of *S. obtusiusculus*, calculated as percent of total dry weight

Biomass component	Content %
	% (g/g dry biomass weight)
Water	3.7
Carbohydrates	33.8
Proteins	48.7
Lipids	8.3
Pigments, secondary metabolites	3.6
Ash	1.9

Relative standard deviation for all given numbers is ≤ ± 2%

### Hydrolysis of *S. obtusiusculus* dry biomass

**Fig. 1:**
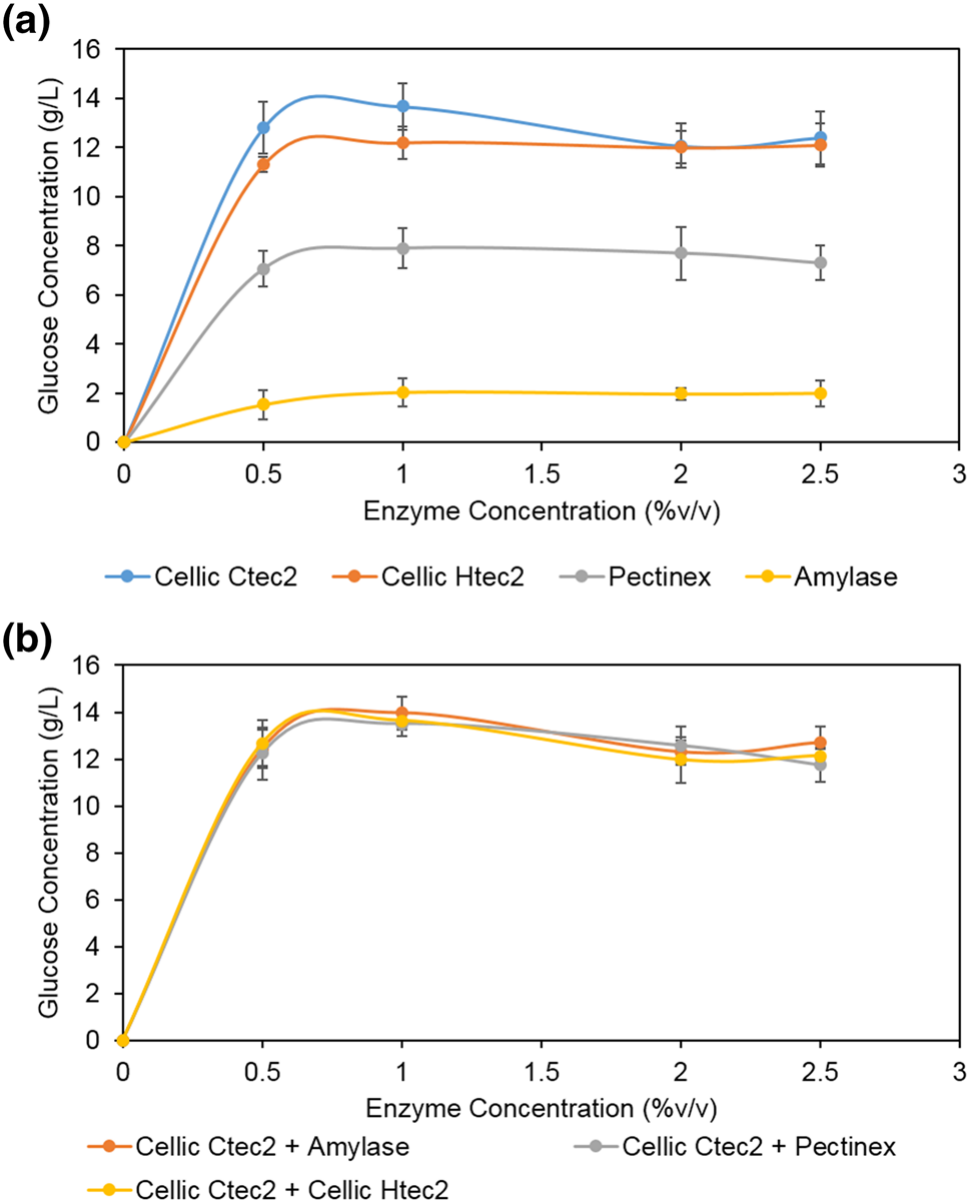
Glucose concentration of SH displayed as a factor of various enzymes mixes and concentrations (**a**). Glucose concentration of SH displayed as a factor of the combination of enzyme mixtures with Cellic CTec 2 (**b**)

Various commercial hydrolase enzyme mixtures were tested for their biomass liquefaction efficiencies including a cellulase mix (Cellic-Ctec2, Novozymes), a hemicellulose-mix (Cellic-Htec2, Novozymes), a pectinase mix (Pectinex, Novozymes), and an amylase mix (Fungamyl, Novozymes). Biomass to glucose conversion ratios from the various enzyme mixtures is presented in Fig. 1a. The cellulase mix (Cellic CTec 2) exhibited the best activity. Glucose monomerization reached a saturation level between 12 and 14 g/L starting from a 50 g algae biomass, which translates to a glucose yield of 0.24-0.28 g/g of DCW. Furthermore, a 1% (v/v) of cellulase mixture Cellic-Ctec2 was combined with varying concentrations of the remaining enzyme mixtures, none of which yielded a significantly better conversion ratio (Fig. 1b).

To assess the efficiency of glucose liberation from the algae biomass, measurements of enzymatic hydrolysis were compared with those of acidic hydrolysis. Table 2 confirms that glucose conversion efficiencies reached (90–100%), with glucose constituting up to two-thirds (g/g of DCW) of the total carbohydrate content of SH.

**Table 2:** A comparison of monosaccharide content % (g/g dry biomass weight) resulting from acidic and enzymatic hydrolysis

Sugar	Acidic hydrolysis	Enzymatic hydrolysis	Conversion
	% (g/g dry biomass weight)	% (g/g dry biomass weight)	Efficiency (%)
Glucose	22	20–22	90–100
Mannose		–	–
Galactose	10	2–2.5	20–25
Rhamnose	~ 1	0	0
Fucose	~ 1	0	0
Ribose	~ 1	0	0

Relative standard deviation for all given numbers is ≤ ± 2%

The combined enzymatic conversion rate of mannose and galactose, which were not distinguished by HPLC, was limited to only 20–25% (g/g DCW).

However, total sugar concentration in the hydrolysate was still relatively low (12–14 g/L glucose) given a carbohydrate content of 34% (g/g DCW). Accordingly, hydrolysis was repeated with higher amounts of biomass retaining a 1% (v/v) concentration of the cellulase mixture. As a result, glucose concentration in SH reached 48 g/L starting with a 200 g *Scenedesmus* dry biomass. This accounts fora glucose yield of 0.24 g/g of DCW, maintaining the high conversion efficiencies of (90–100%). Subsequent experiments were conducted with SH comprising this high glucose concentration (48 g/L).

### Yeast growth and lipid production

**Fig. 2:**
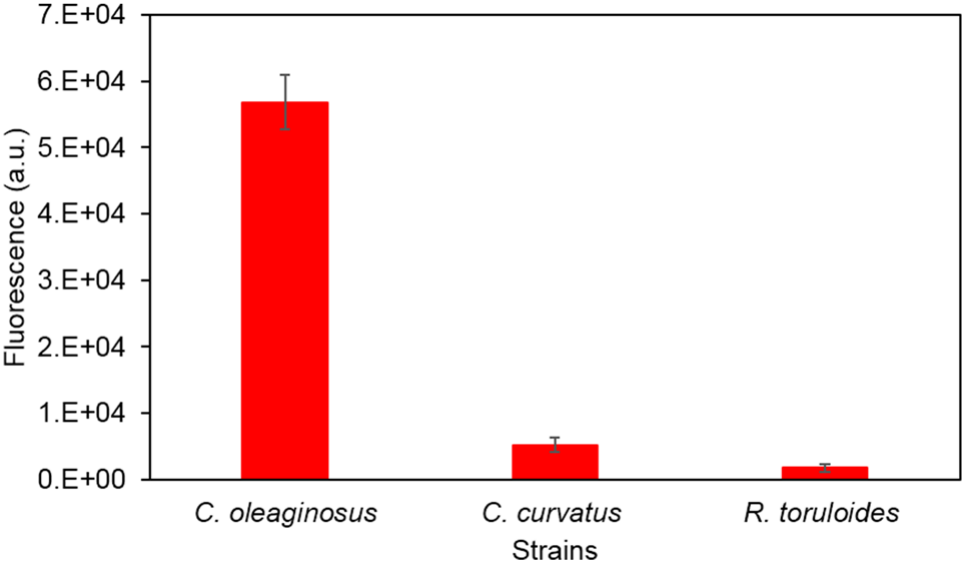
Rapid estimation of lipid contents in *C. oleaginosus*, *C. curvatus* and *R. toruloides* following 4 days cultivation on SH, determined by Nile red assay

High throughput Nile red screening was employed to determine the lipid yield of three oleaginous yeast strains *C. oleaginosus*, *C. curvatus* and *R. toruloides*. Following a 4-day fermentative growth on SH as the sole carbon source, Nile red analysis revealed low lipid content in *C. curvatus* and *R. toruloides*. Contrarily, *C. oleaginosus* was able to accumulate a considerable amount of lipid bodies when cultivated on the same media (Fig. 2).

**Fig. 3:**
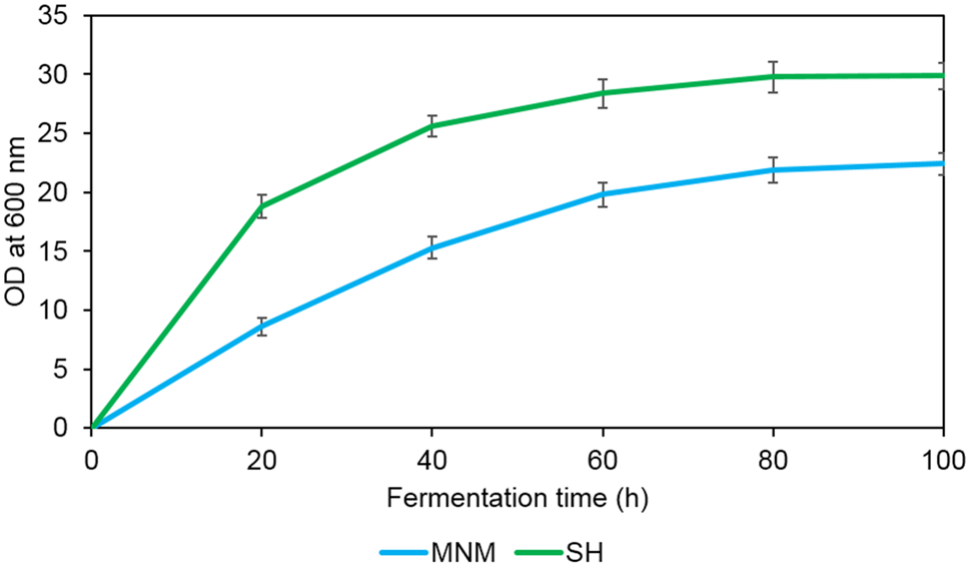
*C. oleaginous* growth trend when grown on MNM and SH over 4 days in a shake flask fermentation

Thus, *C. oleaginosus* underwent subsequent experiments to determine growth rate and lipid absolute quantitation when cultivated on MNM and SH. To evaluate *C. oleaginosus* growth-rate in MNM and SH media, optical density at 600 nm was measured over 4 days (Fig. 3). SH media resulted in highest final growth for *C. oleaginosus* measured at OD_600_ of about 30, compared to OD_600_ of about 22 in MNM.

**Fig. 4:**
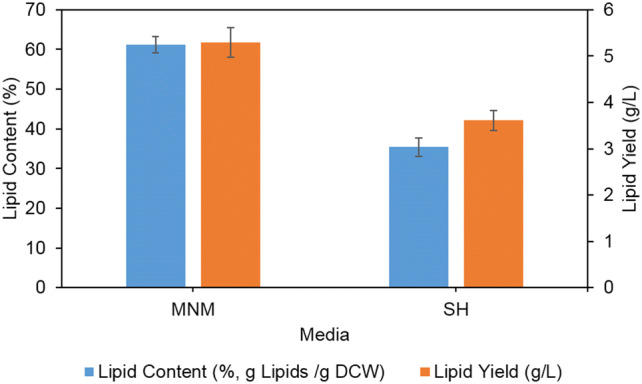
Lipid content (%, g lipid/g DCW) and lipid yield (g/L) of *C. oleaginous* cultivated in MNM and SH media for 4 days in shake flask fermentation

Gravimetric analysis was performed to determine total lipid content in *C. oleaginosus* following fermentation on MNM and SH in shake flasks (Fig. 4). The yeast accumulated nearly 61% and 35% (g lipid/g DCW) lipids when grown on MNM and SH, respectively. After 4 days fermentation, lipid yield in *C. oleaginosus* reached about 5.3 g/L of culture when cultivated on MNM, and 3.6 g/L of culture when cultivated on SH media.

## Discussion

For years, microalgae have been exploited as a source for value-added products, with numerous commercial applications that include enhancing the nutritional value of food and animal feed, as well as being incorporated into cosmetics [44]. The significant properties of microalgae biomass as raw material for microbial cultivation include high carbohydrates contents and lack of recalcitrant lignin [45]. The use of microbes as a platform for lipid and subsequent biofuel and biodiesel production offers: (1) renewability and potential sustainability, (2) requires less labor and fewer raw materials, (3) is easier to scale up, (4) does not compete with edible-plants for land, (5) generates less waste and (6) is not affected by season or climate [46]. Recently, the valorization of seagrass and brown macroalgae biomass as feedstock for *C. oleaginosus* lipid production, in addition to the techno-economic feasibility of the bioprocesses have been conducted in our group [2, 4]. In this study, *S. obtusiusculus* biomass was chosen as feedstock for oily yeast growth, due to its high carbohydrates content 34% (g/g dry biomass weight). In comparison, *Scenedesmus obliquus*, *Chlorella vulgaris*, *Chlamydomonas rheinhardii*, and *Dunaliella salina* algae species exhibit sugar content per dry biomass weight of 10%, 12%, 17% and 32% respectively [44].

Complete chemical hydrolysis (H_2_SO_4_) have been regularly implemented for the production of hydrolysate from lignocellulosic biomass [47, 48]. Lately, two-stage hydrolysis processes starting with mild chemical treatment (dilute sulfuric acid) and followed by enzymatic hydrolysis have gained popularity amongst industrial applications [49]. Corn-stover biomass hydrolysis required a pretreatment of the biomass with 0.5 M NaOH at 80 °C for 75 min [50]. However, these methods often generate inhibitory substances that might hinder or completely abolish the growth of microorganisms cultivated in the resulting hydrolysates. Furfural was found to elongate the lag-phase; while benzoic acid reduced growth rate and biomass yield [51]. Thus, complex detoxification step would be necessary prior to the fermentation, ensuing additional costs and tarnishing the eco-friendly aspect of the biofuel production process [36]. Accordingly, we opted to use a single-step enzymatic approach in this study that would allow efficient hydrolysis of algae biomass without the need for any pretreatment steps. Sterilization of *S. obtusiusculus* biomass was performed in a laboratory-scale autoclave at 120 °C for 15 min to eliminate the microbial contaminants present within the microalgae residue. This not considered as a pretreatment step since hydrolysis of hemicellulose and cellulose only starts at temperatures greater than 150 °C [37]. Efficient saccharification of *S. obtusiusculus* biomass by single-step enzymatic hydrolysis using a cellulase mix was possible. In fact, the Cellic CTec 2 combines a number of different enzymatic activities (exo-, endo-glucanase activity and proteinase activity). The optimal activity was obtained at an industrially relevant concentration of 1% (v/v) at 50.0 °C and pH 5.0 in 50 mM, sodium acetate buffer for 72 h. Quantitative biomass to glucose conversion ratio remained high, even when raising substrate amounts up to 200 g/L. Beyond this point, viscosity was too high for effective hydrolysis. Notably, the diverse heteropolymeric structure of algal cell wall might account for the low conversion efficiency of mannose and galactose [52]. Commonly available enzyme mixtures including Celli-Htec2 (Novozymes), Pectinex (Novozymes) and Fungamyl (Novozymes) failed to liberate total monosaccharides from these structures.

Lipid accumulation in oleaginous yeasts is usually triggered upon excess carbon and nutrient deficiency (e.g., nitrogen phosphate or sulfur). Lipid yields and fatty acid profile vary depending on the type and concentration of the carbon and nitrogen source [1, 7, 53]. Single-step enzymatic hydrolysis generated glucose-rich hydrolysate, the preferred monomeric sugar for microbial fermentation. A 10 KDa cross-filtration was subsequently implemented, and the permeate product (SH), exhibiting now nitrogen limitation and high C/N ratio, was used as fermentation media for high lipid accumulation in yeast.

The oleaginous yeast *Cutaneotrichosporon oleaginous* is able to metabolize a broad monosaccharide spectrum including hexoses and pentoses into intracellular TAGs [23]. This yeast was also able to grow well in a model medium with a carbohydrate mixture that resembled a typical microalgae derived-hydrolysate [54]. In this work, *C. oleaginosus*, as well as *C. curvatus* and *R. toruloides* were cultivated in *S. obtusiusculus* hydrolysate. Most interestingly, in contrast to the other two oleaginous yeasts, high-throughput Nile red analysis indicated that only *C. oleaginosus* was able to accumulate significant amounts of intracellular lipids when grown in SH.

Without any nutritional addition to the hydrolysate (biotin, yeast extract, pure glucose…), SH was utilized as the sole carbon source for lipid production in *C. oleaginosus.* The assessment was conducted on the basis of lipid accumulation. The results were evaluated along with the data from cultivation in the synthetic MNM—a medium known to induce lipid biosynthesis in oleaginous yeasts [55]. *C. oleaginosus* grew faster in SH media, in comparison with MNM. The yeast yielded 61% (g lipid/g DCW) of intracellular lipid when grown on MNM, and about 35% (g lipid/g DCW) when grown on SH. Following fermentation, *C. oleaginosus* achieved total lipid yield of 3.6 g/L when cultivated on SH media. In scaled-up experiments, SH would prove a cost-effective alternative for the relatively expensive synthetic MNM media.

To establish nutrient limitation, the microalgae hydrolysate underwent ultrafiltration thus eliminating proteins and peptides and establishing a high C/N ratio. However, other factors besides nitrogen-limitation could induce lipogenesis in *C. oleaginosus*. Effect of phosphate and sulfur limitation on lipid accumulation in oleaginous yeasts have been previously reported [56]. High C/P ratio prompted high lipid yield in *R. toruloides* even in the presence of excess nitrogen [22]. For future work, soluble phosphates could be precipitated and removed by interaction with metal ions, such as Ca^2+^, Mg^2+^, or Fe^3+^ [57], and the resulting hydrolysate—now exhibiting high C/N and C/P ratios—could allow for even higher for lipid accumulation by *C. oleaginosus.*

*Cutaneotrichosporon oleaginosus* cultivated in *S. obtusiusculus* hydrolysate achieved a high growth rate and accumulated substantial amount of intracellular lipids. Previous research showed that this yeast accumulates lipid in the form of triacylglycerides, with a fatty acid (FA) profile consisting mainly of C16 and C18 FA [58]. Palmitic acid, stearic acid and oleic acid constitute the major raw material for downstream processing and subsequent conversion into green biofuels [59]. Furthermore, chemo-catalytic conversion of lipids produced by *C. oleaginosus* into biodiesel was achieved with a 98.9% w/w recovery [9]. The physical properties of resulting B20 - comparable to Soybean B20 - meet the ASTM requirements [9]. This FA profile makes SCOs from *C. oleaginosus* a suitable alternative for plant and vegetable oils.

## Conclusion

This study demonstrated that *Scenedesmus obtusiusculus* biomass could be valorized as a substrate for microbial lipid production. A single-step enzymatic hydrolysis was implemented that efficiently released monomeric sugars from the biomass without the need for any pretreatment. This approach alleviated the need for detoxification steps, reduced upstream processing costs and maintained the eco-friendly aspect of biofuel production. The oleaginous yeast *C. oleaginosus* was able to grow fast and accumulate 3.6 g/L of lipids when cultivated on the microalgae hydrolysate, and the resulting microbial oil could be converted to high-grade biodiesel. Microalgae biomass offer value-added biofuel yield potential as compared to terrestrial plantation; biomass-to-fuel conversion processes are improved by necessitating no agricultural land, alleviating direct competition with food security and requiring low water and resource demand. Furthermore, the integration of yeast and algae species in a single SCO platform towards “zero concepts” with respect to emission and excess resources has recently been reported. In one study, the oleaginous microalgae *Phaeodactylum tricornutum* was supplemented with CO_2_ supplied from the oleaginous yeast *C. curvatus* in a co-fermentation approach [32]. In another study focused on the holistic valorization of unexploited marine biomass, a waste-free, microbial oil-centered cyclic bio-refinery approach integrated the production of yeast lipids and animal feed with precious metal biosorbents [4]. In that respect, the algal effluent resulting from the ultra-filtration of the algae hydrolysate should be further characterized and profiled for possible added-value in the process described in this study.

## Data Availability

All datasets generated for this study are included in the manuscript.

## References

[1] Awad D, Bohnen F, Mehlmer N, Brueck T (2019). Multi-factorial-guided media optimization for enhanced biomass and lipid formation by the oleaginous yeast *Cutaneotrichosporon oleaginosus*. Front Bioeng Biotechnol.

[2] Masri MA, Younes S, Haack M, Qoura F, Mehlmer N, Brück T (2018). A seagrass-based biorefinery for generation of single-cell oils for biofuel and oleochemical production. Energy Technol.

[3] Knothe G (2008). “Designer” biodiesel: optimizing fatty ester composition to improve fuel properties. Energy Fuels.

[4] Masri MA, Jurkowski W, Shaigani P, Haack M, Mehlmer N, Brück T (2018). A waste-free, microbial oil centered cyclic bio-refinery approach based on flexible macroalgae biomass. Appl Energy.

[5] Smith VH, Sturm BS, Billings SA (2010). The ecology of algal biodiesel production. Trends Ecol Evol.

[6] Kyle DJ (2010). Future development of single cell oils. Single cell oils.

[7] Ratledge C, Wynn JP (2002). The biochemistry and molecular biology of lipid accumulation in oleaginous microorganisms. Adv Appl Microbiol.

[8] Bracharz F, Beukhout T, Mehlmer N, Bruck T (2017). Opportunities and challenges in the development of Cutaneotrichosporon oleaginosus ATCC 20509 as a new cell factory for custom tailored microbial oils. Microb Cell Fact.

[9] McCurdy AT, Higham AJ, Morgan MR, Quinn JC, Seefeldt LC (2014). Two-step process for production of biodiesel blends from oleaginous yeast and microalgae. Fuel.

[10] Coradetti ST, Pinel D, Geiselman GM, Ito M, Mondo SJ, Reilly MC, Cheng Y-F, Bauer S, Grigoriev IV, Gladden JM, Simmons BA, Brem RB, Arkin AP, Skerker JM (2018). Functional genomics of lipid metabolism in the oleaginous yeast Rhodosporidium toruloides. eLife.

[11] Kosa M, Ragauskas AJ (2011). Lipids from heterotrophic microbes: advances in metabolism research. Trends Biotechnol.

[12] Meng X, Yang J, Xu X, Zhang L, Nie Q, Xian M (2009). Biodiesel production from oleaginous microorganisms. Renew Energy.

[13] Rakicka M, Lazar Z, Dulermo T, Fickers P, Nicaud JM (2015). Lipid production by the oleaginous yeast Yarrowia lipolytica using industrial by-products under different culture conditions. Biotechnol Biofuels.

[14] Azambuja SPH, Bonturi N, Miranda EA, Gombert AK (2018). Physiology and lipid accumulation capacity of different <em> *Yarrowia lipolytica* </em> and <em> *Rhodosporidium toruloides* </em> strains on glycerol. bioRxiv.

[15] Rakicka M, Lazar Z, Dulermo T, Fickers P, Nicaud JM (2015). Lipid production by the oleaginous yeast Yarrowia lipolytica using industrial by-products under different culture conditions. Biotechnol Biofuels.

[16] Wang R, Wang J, Xu R, Fang Z, Liu A (2014). Oil production by the oleaginous yeast *Lipomyces starkeyi* using diverse carbon sources. BioResources.

[17] Moon NJ, Hammond E, Glatz BA (1978). Conversion of cheese whey and whey permeate to oil and single-cell protein1. J Dairy Sci.

[18] Evans CT, Ratledge C (1983). A comparison of the oleaginous yeast, *Candida curvata*, grown on different carbon sources in continuous and batch culture. Lipids.

[19] Liang Y, Jarosz K, Wardlow AT, Zhang J, Cui Y (2014). Lipid production by *Cryptococcus curvatus* on hydrolysates derived from corn fiber and sweet sorghum bagasse following dilute acid pretreatment. Appl Biochem Biotechnol.

[20] Chi Z, Ahring BK, Chen S (2012). Oleaginous yeast *Cryptococcus curvatus* for biofuel production: ammonia’s effect. Biomass Bioenerg.

[21] Iassonova DR (2009). Lipid synthesis and encapsulation by *Cryptococcus curvatus*.

[22] Wu S, Hu C, Zhao X, Zhao ZK (2010). Production of lipid from *N*-acetylglucosamine by *Cryptococcus curvatus*. Eur J Lipid Sci Technol.

[23] Gujjari P, Suh S-O, Coumes K, Zhou JJ (2011). Characterization of oleaginous yeasts revealed two novel species: trichosporon *Cacaoliposimilis* sp. nov. and *Trichosporon oleaginosus* sp. nov. Mycologia.

[24] Masri MA, Garbe D, Mehlmer N, Brück TB (2019). A sustainable, high-performance process for the economic production of waste-free microbial oils that can replace plant-based equivalents. Energy Environ Sci.

[25] Ageitos JM, Vallejo JA, Veiga-Crespo P, Villa TG (2011). Oily yeasts as oleaginous cell factories. Appl Microbiol Biotechnol.

[26] Bai A, Popp J, Pető K, Szőke I, Harangi-Rákos M, Gabnai Z (2017). The significance of forests and algae in CO_2_ balance: a hungarian case study. Sustainability.

[27] Bhola V, Swalaha F, Ranjith Kumar R, Singh M, Bux F (2014). Overview of the potential of microalgae for CO_2_ sequestration. Int J Environ Sci Technol.

[28] Sayre R (2010). Microalgae: the potential for carbon capture. Bioscience.

[29] Koutinas AA, Chatzifragkou A, Kopsahelis N, Papanikolaou S, Kookos IK (2014). Design and techno-economic evaluation of microbial oil production as a renewable resource for biodiesel and oleochemical production. Fuel.

[30] Guo M, Cheng S, Chen G, Chen J (2019). Improvement of lipid production in oleaginous yeast *Rhodosporidium toruloides* by ultraviolet mutagenesis. Eng Life Sci.

[31] André A, Chatzifragkou A, Diamantopoulou P, Sarris D, Philippoussis A, Galiotou-Panayotou M, Komaitis M, Papanikolaou S (2009). Biotechnological conversions of bio-diesel-derived crude glycerol by Yarrowia lipolytica strains. Eng Life Sci.

[32] Dillschneider R, Schulze I, Neumann A, Posten C, Syldatk C (2014). Combination of algae and yeast fermentation for an integrated process to produce single cell oils. Appl Microbiol Biotechnol.

[33] Achinas S, Achinas V, Euverink GJW (2017). A technological overview of biogas production from biowaste. Engineering.

[34] Htet AN, Noguchi M, Ninomiya K, Tsuge Y, Kuroda K, Kajita S, Masai E, Katayama Y, Shikinaka K, Otsuka Y, Nakamura M, Honda R, Takahashi K (2018). Application of microalgae hydrolysate as a fermentation medium for microbial production of 2-pyrone 4,6-dicarboxylic acid. J Biosci Bioeng.

[35] Wirth R, Lakatos G, Böjti T, Maróti G, Bagi Z, Kis M, Kovács A, Ács N, Rákhely G, Kovács KL (2015). Metagenome changes in the mesophilic biogas-producing community during fermentation of the green alga *Scenedesmus obliquus*. J Biotechnol.

[36] Huang H, Guo X, Li D, Liu M, Wu J, Ren H (2011). Identification of crucial yeast inhibitors in bio-ethanol and improvement of fermentation at high pH and high total solids. Bioresour Technol.

[37] Silveira MHL, Morais ARC, da Costa Lopes AM, Olekszyszen DN, Bogel-Łukasik R, Andreaus J, Pereira Ramos L (2015). Current pretreatment technologies for the development of cellulosic ethanol and biorefineries. Chemsuschem.

[38] Shetlar M, Masters YF (1957). Use of thymol-sulfuric acid reaction for determination of carbohydrates in biological material. Anal Chem.

[39] Kjeldhal J (1883). A new method for estimation of nitrogen in organic compounds. Z Anal Chem.

[40] Folch J, Lees M, Sloane-Stanley G (1957). A simple method for the isolation and purification of total lipids from animal tissues. J Biol Chem.

[41] Chemists AoOA (1990) Official methods of analysis: changes in official methods of analysis made at the annual meeting. Supplement, vol 15. Association of Official Analytical Chemists,

[42] Suutari T, Priha P, Laakso S (1993) Temprature shifts in regulation of lipids accumulated by *Lippmyces starkeyi*, p. 891–894

[43] Sitepu I, Ignatia L, Franz A, Wong D, Faulina S, Tsui M, Kanti A, Boundy-Mills K (2012). An improved high-throughput Nile red fluorescence assay for estimating intracellular lipids in a variety of yeast species. J Microbiol Methods.

[44] Spolaore P, Joannis-Cassan C, Duran E, Isambert A (2006). Commercial applications of microalgae. J Biosci Bioeng.

[45] Posten C (2009). Design principles of photo-bioreactors for cultivation of microalgae. Eng Life Sci.

[46] Li Q, Du W, Liu D (2008). Perspectives of microbial oils for biodiesel production. Appl Microbiol Biotechnol.

[47] Morales-delaRosa S, Campos-Martin JM, Fierro JL (2014). Optimization of the process of chemical hydrolysis of cellulose to glucose. Cellulose.

[48] Tsigie YA, Wang C-Y, Kasim NS, Diem Q-D, Huynh L-H, Ho Q-P, Truong C-T, Ju Y-H (2012). Oil production from *Yarrowia lipolytica* Po1g using rice bran hydrolysate.

[49] Zhang Y, Zhang M, Reese RA, Zhang H, Xu B (2016). Real-time single molecular study of a pretreated cellulose hydrolysis mode and individual enzyme movement. Biotechnol Biofuels.

[50] Gong Z, Shen H, Yang X, Wang Q, Xie H, Zhao ZK (2014). Lipid production from corn stover by the oleaginous yeast *Cryptococcus curvatus*. Biotechnol Biofuels.

[51] Zha Y, Muilwijk B, Coulier L, Punt PJ (2012). Inhibitory compounds in lignocellulosic biomass hydrolysates during hydrolysate fermentation processes. J Bioprocess Biotechniq.

[52] Takeda H (1988). Classification of Chlorella strains by cell wall sugar composition. Phytochemistry.

[53] Granger LM, Perlot P, Goma G, Pareilleux A (1993). Efficiency of fatty acid synthesis by oleaginous yeasts: prediction of yield and fatty acid cell content from consumed C/N ratio by a simple method. Biotechnol Bioeng.

[54] Meo A, Priebe XL, Weuster-Botz D (2017). Lipid production with Trichosporon oleaginosus in a membrane bioreactor using microalgae hydrolysate. J Biotechnol.

[55] L-j MA, H-m WANG, Y-f WEN, H-l JIANG, D-h XUE (2008). Optimized culture medium and fermentation conditions for lipid production with filamentous fungus. J Changchun Univ Technol.

[56] Zhang G, French WT, Re Hernandez, Hall J, Sparks D, Holmes WE (2011). Microbial lipid production as biodiesel feedstock from *N*-acetylglucosamine by oleaginous microorganisms. J Chem Technol Biotechnol.

[57] Jenkins D, Ferguson JF, Menar AB (1971). Chemical processes for phosphate removal. Water Res.

[58] Kourist R, Bracharz F, Lorenzen J, Kracht ON, Chovatia M, Daum C, Deshpande S, Lipzen A, Nolan M, Ohm RA (2015). Genomics and transcriptomics analyses of the oil-accumulating basidiomycete yeast Trichosporon oleaginosus: insights into substrate utilization and alternative evolutionary trajectories of fungal mating systems. MBio.

[59] Zhao C, Brück T, Lercher JA (2013). Catalytic deoxygenation of microalgae oil to green hydrocarbons. Green Chem.

